# The mental health implications of informal care receipt stability among older adults with persistent care needs

**DOI:** 10.1093/geronb/gbaf154

**Published:** 2025-08-21

**Authors:** Yezhen Li, Marco Angrisani, Jinkook Lee

**Affiliations:** Center for Economic and Social Research, University of Southern California, Los Angeles, California, United States; Center for Economic and Social Research, University of Southern California, Los Angeles, California, United States; Department of Economics, University of Southern California, Los Angeles, California, United States; Center for Economic and Social Research, University of Southern California, Los Angeles, California, United States; Department of Economics, University of Southern California, Los Angeles, California, United States

**Keywords:** Long-term care, Functional limitations, Depressive symptoms

## Abstract

**Objectives:**

Despite growing scholarship on the mental health consequences of informal care receipt, little is known about how longitudinal dynamics of informal care—specifically, the stability of informal care—shape older adults’ psychological well-being. This study introduces a measure of informal care receipt stability and investigates 1) how the stability of informal care receipt predicts depressive symptoms among older adults with persistent care needs, and 2) how it moderates the associations between functional limitations, i.e., activities of daily living (ADL) and instrumental activities of daily living (IADL) limitations, and depressive symptoms.

**Methods:**

We used data from the 2010–2018 waves of the Health and Retirement Study (*n* = 4,160 respondents; 8,332 person-year observations). The analysis employed mixed-effect models to predict depressive symptoms.

**Results:**

Receiving stable informal care is associated with significantly fewer depressive symptoms than not receiving informal care. Stable informal care also weakens the negative association between IADL limitations and depressive symptoms, though it does not play a similar role for ADL limitations. Additional analyses demonstrate that informal care stability is particularly important for the mental health of older adults who receive care from close family members, i.e., spouses and children.

**Discussion:**

These findings imply that informal care stability serves as a measure of care quality through a longitudinal lens, especially for older adults receiving care from their closest kin. Policies encouraging caregiving commitments from core family members and those facilitating care coordination among extended family and nonrelative caregivers are crucial for the mental health of long-term care recipients.

Informal caregivers, or those providing unpaid care to people in their personal networks, have served as the primary source of assistance to older U.S. adults with functional limitations in recent decades (Van Houtven et al., 2020). A growing body of literature has examined how receiving informal care shapes individuals’ physical and mental health, offering important insights into care quality (Coe et al., 2021). However, little is known about how the dynamics of informal care receipt *over time* impact the well-being of older adults with long-term care needs. Understanding the longitudinal patterns of informal care receipt, particularly their roles in older adults’ well-being, may have crucial implications for policies aimed at sustaining long-term care for individuals with persistent needs (Reinhard et al., 2023).

Using data from the Health and Retirement Study, this study introduces a measure of informal care receipt stability and investigates 1) how informal care stability predicts depressive symptoms among older adults with persistent care needs, and 2) how it alleviates the harmful mental health effects of activities of daily living (ADL) limitations and instrumental activities of daily living (IADL) limitations. We find that receiving stable informal care over time is associated with lower levels of depressive symptoms than not receiving informal care. Stable informal care receipt also weakens the negative association between IADL limitations and depressive symptoms, though it does not play a similar role for ADL limitations. In addition, informal care stability is particularly important for the mental health of older adults receiving care from their spouses and children. We conclude by discussing the implications for understanding care quality from a longitudinal perspective and long-term care policies.

## Background

### Informal care receipt and mental health

Compared with the large literature on the mental health of informal caregivers, research examining the psychological consequences of receiving informal care is relatively limited (Coe et al., 2021). In this vein, several studies have found that older adults receiving informal care fared worse in mental health and quality-of-life measures, such as subjective well-being and life satisfaction, compared to those receiving no care (Kwak et al., 2014; Lin & Wu, 2011; Shang & Patterson, 2024; Zwar et al., 2019).

Though scholars have raised concerns about the endogeneity of care receipt that drives these findings (Coe et al., 2021; Mosca et al., 2012; Pristavec & Luth, 2020), empirical evidence has documented the complex mechanisms through which informal care influences recipients’ mental health. For example, having a caregiver may heighten older adults’ negative perceptions of aging and diminished sense of control, which may elevate depressive symptoms (Brown, 2007; Kwak et al., 2014; Zwar et al., 2023). Others have highlighted the positive aspects of care use, including the promotion of social integration (Floridi et al., 2024). Care quality also plays a key role, as insufficient care and poor relationships with caregivers may worsen mental health (Swinkels et al., 2025; Wolff & Agree, 2004). In addition, the psychological consequences of informal care may vary by caregiver type, though the evidence is mixed. For instance, Brown (2007) suggested that older adults with family caregivers reported worse mental health than those using formal care, whereas Swinkels et al. (2025) found the opposite pattern.

Meanwhile, scholars emphasized the role of informal care receipt in buffering the deleterious mental health effects of functional limitations (Carr et al., 2017; Chan et al., 2010). Functional disabilities cause substantial distress by impeding individuals from carrying out basic daily tasks, and their adverse mental health consequences may be alleviated by instrumental support from others (Forder et al., 2018; Silverstein & Bengtson, 1994). In particular, Chan et al. (2010) found that only the receipt of informal care, rather than formal care, attenuates the negative association between functional disabilities and mental health. However, these studies did not differentiate between ADL and IADL limitations. ADL limitations are generally recognized as more severe disabilities, which cause greater psychological distress that can be difficult to alleviate through informal care (Spector et al., 1987). In contrast, IADL limitations refer to difficulties with more complex, instrumental tasks in one’s daily routine, and addressing these limitations requires individual-specific support (Bruderer-Hofstetter et al., 2022; Nygård, 2003). Considering the differences in the two types of limitations, whether informal care buffers their mental health effects merits separate examination.

More importantly, scholars have yet to fully understand how longitudinal dynamics of informal care receipt shape care recipients’ mental health. The present study focuses on informal care stability as a dimension of care receipt dynamics. The provision of sustainable community–based care has been a key priority of long-term care policies (Allen et al., 2012). Examining the psychological implications of informal care stability advances the understanding of care quality through a temporal lens. We ask: 1) Does the receipt of stable and unstable informal care predict depressive symptoms, compared to no informal care receipt, among older adults with persistent care needs? 2) Does informal care stability moderate the associations between functional limitations, including ADL and IADL limitations, and depressive symptoms?

### The stability of informal care receipt

We define the stability of informal care receipt as receiving informal care from the same caregiver(s), e.g., spouse, children, other relatives, or nonrelatives, over time for individuals with persistent care needs; conversely, informal care instability may occur through the loss, addition, or transitions between informal caregivers. To date, empirical evidence on informal care stability remains scarce. Early scholarship suggested that changes in informal care primarily resulted from respondents’ health and functional conditions (Kelman et al., 1994). Allen et al. (2012) found that risks of care transitions were a function of caregiver–care recipient relationship type (core family members, other relatives, vs non-kin) and their gender match/mismatch. Meanwhile, core family caregivers are often more attuned to older adults’ needs and preferences, which facilitates high-quality care (Allen & Ciambrone, 2003; Shang & Patterson, 2024). Allen and colleagues (2012) implied that informal care stability may serve as a benchmark of care quality, though they did not empirically test this implication (e.g., examining the well-being consequences).

Even less is known about how informal care stability shapes recipients’ mental health. As an exception, Di Novi et al. (2023) found that disruptions of informal care receipt during the COVID-19 pandemic contributed to older adults’ depression. However, the study only focused on experiences of losing care, thus overlooking the implications of care transition. There are good reasons to speculate that caregiver transitions dampen the mental health benefits of care. For example, David and Kim (2018) found that among formal care recipients, handoffs between home-health nurses led to higher risks of hospital readmission. Social network research also documented that later–life personal network stability was linked with better physical and psychological well-being, through promoting personal trust and enabling high-quality social support (Cornwell & Laumann, 2015; Schwartz & Litwin, 2017).

Building on the existing evidence, we posit that the stability of informal care constitutes a measure of care quality that has important mental health implications. Receiving stable informal care allows caregivers to develop strong relationships with their care recipients, which promotes the quality of their instrumental support. By contrast, the mental health benefits of informal care may be diminished by care instability, as care interruptions may compound recipients’ feelings of relationship loss, and care transitions hamper close bonds between caregivers and care recipients. The mental health consequences of informal care stability may be especially pronounced for older adults receiving care from spouses and children, who tend to better understand older adults’ personal needs. The loss of these caregivers may disrupt high-quality care delivery and intensify feelings of abandonment among care recipients.

### The role of informal care stability in the relationships between functional limitations and depressive symptoms

Stable receipt of informal care may play an important role in buffering limitation-related distress (Chan et al., 2010). This study investigates the moderation effects of informal care stability on the associations between current functional limitations, measured through the number of ADL or IADL limitations, and depressive symptoms (Na & Streim, 2017; Xiao et al., 2022).

The moderation effect of stable informal care may vary by limitation type. On the one hand, informal care stability could be more important for buffering ADL limitation-induced distress. ADL limitations impose substantial challenges in individuals’ fundamental aspects of their daily lives. Losing support over essential daily tasks may introduce feelings of isolation, frustration, and diminished self-esteem (Na & Streim, 2017). Additionally, as ADLs involve private facets of daily lives, frequent caregiver transitions may heighten older adults’ feelings of privacy invasion and diminished self-control (Boumans et al., 2018; Roe et al., 2001). However, ADL limitations constitute severe disabilities leading to greater dysfunction and loss of independence among older adults (Spector et al., 1987). The inability to perform the most essential self-care tasks, coupled with the heightened disability status, may cause significant distress that even consistent informal care can hardly mitigate.

On the other hand, stable informal care might play a more crucial role in the associations between IADL limitations and depressive symptoms. IADLs are often individual-specific activities that depend on individuals’ personal needs (e.g., grocery plans, financial conditions, and food tastes) and a wide array of sociocontextual factors (Bruderer-Hofstetter et al., 2022; Moon et al., 2024; Nygård, 2003). Stable informal care enables the caregiver to better understand the care recipient’s preferences, contributing to more efficient care delivery (Allen et al., 2012). By contrast, caregiver transitions may preclude a trustful caregiver–care recipient relationship; the disrupted support may fail to alleviate depressive symptoms induced by IADL limitations (David & Kim, 2018).

## Method

### Data

The present study uses data from the Health and Retirement Study (HRS). HRS is sponsored by the National Institute on Aging (Grant No. NIA U01AG009740) and conducted by the University of Michigan. It is a nationally representative, longitudinal survey of adults aged 50 years and older, conducted biennially since 1992. HRS collects information about respondents’ care needs due to functional limitations, as well as formal and informal care receipt. Respondents were considered in need of care if they reported at least one of the six ADL limitations (difficulties in dressing, walking across a room, bathing, eating, getting in and out of bed, and using the toilet) or five IADL limitations (difficulties in preparing meals, grocery shopping, making phone calls, taking medications, and managing money). Only these respondents received questions on care receipts.

This study relies on the data obtained from the 2010s, using the 2010, 2012, 2014, 2016, and 2018 HRS waves. As the long-term trends in informal care are affected by shifts in caregiving norms (Janus & Doty, 2017), we limit the time span to a decade to study care dynamics. We restrict the sample to adults aged 50+ with *persistent care needs*, i.e., reporting at least one (I)ADL limitation during two consecutive waves. Respondents who reported limitations during zero or one wave and those with nonconsecutive limitation reports were excluded from the sample.

As detailed later, the analyses used longitudinal models to study the mental health implications of informal care stability across two timepoints (hereafter, *t*_1_ and *t*_2_), separated by a 2-year interval. The analytic sample included person-year observations from respondents who reported at least one ADL or IADL limitation at both timepoints. Observations from a proxy interview (i.e., conducted with a family member) at *t*_2_ were excluded because information about participants’ *t*_2_ depressive symptoms was unavailable. Finally, the study limits its scope to community-dwelling older adults. Person-year observations where respondents were interviewed in a health facility at either *t*_1_ or *t*_2_ were not included in the sample. The final sample consists of 4,160 respondents with 8,332 person-year observations.

### Measures

#### Dependent variable

An index based on the shortened version of the Center for Epidemiologic Studies Depression Scale measures *depressive symptoms*. The index, ranging from 0 to 8, is the sum of eight items on whether the respondent felt depressed, happy, lonely, sad, everything was an effort, their sleep was restless, they couldn’t get going, and enjoyed life much of the time over the week before the interview. The items were (re)coded such that higher values indicate greater depressive symptoms.

#### Functional limitation measures

Two variables measure respondents’ limitations: the *number of ADL limitations* (0–6) and the *number of IADL limitations* (0–5). Both variables are measured at *t*_2_.

#### Informal care stability measure

**Figure 1.**
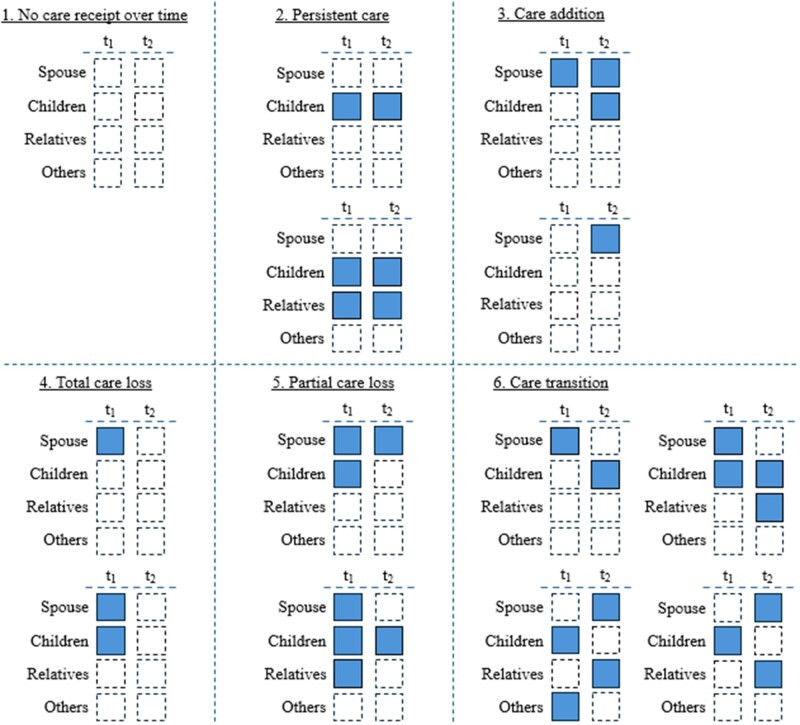
The six categories of the informal care stability measure. Individuals are assumed to need care (i.e., report at least one ADL or IADL limitation) at both *t*_1_ and *t*_2_. A filled square with solid boundaries indicates that the individual received informal care from the corresponding source at the corresponding time point; a blank square with dashed boundaries indicates otherwise. The examples in each category are not exhaustive; see the *Informal Care Stability Measure* section of the main manuscript for full descriptions. ADL = activities of daily living; IADL = instrumental activities of daily living.

We develop a measure of *informal care stability*, consisting of six categories. During each wave, HRS asked respondents with care needs whether they received informal care from four sources: spouse, children, other relatives, and/or nonrelatives (e.g., friends and neighbors). Figure 1 illustrates the categories that capture changes in informal care between *t*_1_ and *t*_2_, among respondents with care needs at both timepoints. They are as follows:

1. No care receipt over time: This category consists of respondents who did not report any informal care receipt at both *t*_1_ and *t*_2_. It constitutes the reference category.
2. Persistent care: The category is comprised of respondents who reported at least one informal care source at *t*_1_, and retained all source(s) at *t*_2_, without adding any new source.
3. Care addition: The category consists of those who added at least one informal care source at *t*_2_, compared to *t*_1_, without losing any informal care source.
4. Total care loss: The category comprises respondents who reported at least one source at *t*_1_, and no informal care source at *t*_2_.
5. Partial care loss: This category consists of those who reported at least two informal care sources at *t*_1_, and then lost some, but not all, sources at *t*_2_, without adding any new sources.
6. Care transition: The category is comprised of respondents who experienced both loss and addition of informal care sources between two timepoints. The most common form was the replacement of one informal care source at *t*_1_ with another source at *t*_2_. There were more complex forms of care transition, through the loss and addition of multiple care sources. They were combined into one category to enhance model parsimony.

In supplemental analysis, we employed two more detailed measures of informal care stability. The first one disaggregated these categories by the type of informal care source at *t*_1_ (spouse only, children only, other relatives only, nonrelatives only, multiple sources, or no care at *t*_1_). The second one disaggregated the partial care loss category by the type of source retained at *t*_2_ (spouse only, children only, other relatives only, nonrelatives only, or multiple but not all sources).

#### Covariates

The analysis accounts for respondents’ sociodemographic characteristics, including *age* (linear and quadratic terms), *female*, *race/ethnicity* (non-Hispanic White, Black, Latino, and others), *educational attainment* (less than high school, high-school graduate, some college, and BA degree), and *log total household wealth at t_1_*.

An individual’s health conditions could be the cause of care receipt instability (Kelman et al., 1994). The analysis controls for *depressive symptoms at t_1_*, a four-category measure (0–1, 2–4, 5–8, representing three tertiles; and missing due to proxy at *t*_1_) based on respondents’ *t*_1_ CES-D scores. Another control measures respondents’ *number of chronic conditions at t_1_*, including stroke, heart disease, hypertension, diabetes, cancer, lung disease, and arthritis. Furthermore, we control for the *number of ADL limitations at t_1_* and the *number of IADL limitations at t_1_*, which are determinants of both care instability and later depressive symptoms (Lin & Wu, 2011).

Social capital is another factor shaping both mental health and informal care receipt. *Marital status at t_1_* is a four-category measure (partnered/married, separated/divorced, widowed, and never married). Another binary indicator measures whether the *respondent lived alone at t_1_*. *Number of proximate children at t_1_* captures the number of respondents’ children who lived within 10 miles of their residence.

Later-life relationship changes, especially those concerning marriages, could shape both informal care instability and mental health (Cornwell & Qu, 2024; Peña-Longobardo et al., 2021). The analysis includes two indicators of *spousal death between t_1_ and t_2_* and *divorce between t_1_ and t_2_*.

As formal care could be a potential substitution for informal care, the analysis also controls for *receipt of formal care*, at both *t*_1_ and *t*_2_. Finally, we include an indicator of *rural residence* and *year* fixed effects.

### Method

The main analysis consists of two parts. First, we examined the person-year level descriptive statistics, stratified by informal care stability categories. *t*-tests of the variables involved in analyses were conducted between observations with no informal care over time (the reference category) and those with other states of care stability. Additionally, we examined the descriptive patterns of informal care stability by respondents’ informal care source at *t*_1_, as well as the distributions of partial care loss observations by the type of care source retained at *t*_2_.

Second, we used mixed-effect linear models to predict depressive symptoms at *t*_2_, with an individual-level random intercept. The analysis consisted of three models. The first model examined the association between informal care stability and depressive symptoms, conditional on covariates. The second and third models examined the moderating roles of informal care stability in the association between functional limitations and depressive symptoms, by including interaction terms between informal care stability and numbers of ADL/IADL limitations at *t*_2_. To aid interpretations, we plotted predicted depressive symptoms at *t*_2_ by the number of (I)ADL limitations and informal care stability.

In *supplemental analyses*, we examined whether the associations between informal care stability and depressive symptoms varied by initial caregiver type. For partial care loss, we analyzed whether its mental health implications varied by the type of care source retained. We also analyzed heterogeneity by individuals’ limitation progressions and their formal care receipts. The improvements in limitation progressions, as well as the transition to formal care, may inform individuals to reduce their informal care receipt, thereby bringing caveats to the proposed negative associations between informal care instability and mental health (Allen et al., 2012; Kelman et al., 1994). Moreover, we examined potential sample attrition bias by incorporating inverse probability weights in main analysis models.

Missing data was addressed using multiple imputations by chained equations, with 10 imputed datasets. As HRS does not provide longitudinal weights, the models are not weighted.

## Results

### Main analysis

Table 1 presents descriptive statistics stratified by categories of the informal care stability measure. The most common pattern of informal care receipt within a 2-year interval was persistent care, comprising 34.2% of cases, followed by no informal care over time (23.0%). However, experiences of losing care were not rare, as total and partial care losses together accounted for around 19% of person-year observations.

**Table 1.** Person-year level descriptive statistics, stratified by informal care stability.

Variables	Informal care stability category						*t*-tests
	No care receipt over time	Persistent care	Care addition	Total care loss	Partial care loss	Care transition	
**Number of person-years**	1,913	2,851	1,681	931	626	330	
**% Person-years in the overall sample**	23.0	34.2	20.2	11.2	7.5	4.0	
**Depressive symptoms at *t*_2_, mean (*SD*)**	2.92 (.06)	3.12 (.05)	3.35 (.06)	3.11 (.08)	3.44 (.10)	3.78 (.14)	a,b,d,e
**Number of ADL limitations at *t*_2_, mean (*SD*)**	1.56 (.03)	2.20 (.03)	2.23 (.04)	1.64 (.05)	2.51 (.08)	2.31 (.10)	a,b,d,e
**Number of IADL limitations at t_2_, mean (*SD*)**	.52 (.02)	1.90 (.03)	1.68 (.03)	.69 (.03)	1.88 (.05)	1.95 (.07)	a,b,c,d,e
**Age, mean (*SD*)**	69.86 (.25)	72.03 (.22)	70.93 (.29)	68.17 (.37)	69.16 (.47)	67.79 (.65)	a,b,c,e
**Gender, %**							
** Female**	60.8	66.9	61.6	61.5	68.8	72.7	a,d,e
** Male**	39.2	33.1	38.4	38.5	31.2	27.3	a,d,e
**Race, %**							
** Non-Hispanic White**	58.0	51.2	51.9	48.1	44.6	44.2	a,b,c,d,e
** Black**	25.1	24.8	25.6	26.5	30.0	32.1	d,e
** Latino**	13.9	20.2	18.6	20.2	21.7	21.8	a,b,c,d,e
** Other**	3.0	3.8	3.9	5.2	3.7	1.8	c
**Educational attainment, %**							
** Less than high school**	19.9	35.5	30.5	29.3	35.0	34.8	a,b,c,d,e
** High school graduate**	35.0	34.5	34.6	34.2	29.1	33.0	d
** Some college**	27.9	19.9	23.5	24.8	24.6	23.3	a,b
** BA degree**	17.2	10.0	11.4	11.7	11.3	8.8	a,b,c,d,e
**Logged household wealth at *t*_1_, mean (*SD*)**	15.38 (.00)	15.38 (.00)	15.38 (.00)	15.38 (.00)	15.37 (.00)	15.36 (.00)	c,d,e
**Depressive symptoms at *t*_1_, %**							
** 0–1**	38.4	31.4	33.5	31.5	24.9	24.5	a,b,c,d,e
** 2–4**	35.2	36.2	32.7	33.8	36.7	35.2	
** 5–8**	26.1	30.2	32.6	33.1	35.3	39.1	a,b,c,d,e
** Missing due to proxy interview**	0.2	2.1	1.2	1.6	3.0	1.2	a,b,c,d,e
**Number of chronic conditions at *t*_1_, mean (*SD*)**	2.55 (.03)	2.95 (.03)	2.86 (.03)	2.72 (.05)	3.11 (.06)	2.90 (.07)	a,b,c,d,e
**Number of ADL limitations at *t*_1_, mean (*SD*)**	1.47 (.02)	2.06 (.03)	1.75 (.04)	1.86 (.05)	2.57 (.07)	2.10 (.09)	a,b,c,d,e
**Number of IADL limitations at *t*_1_, mean (*SD*)**	.50 (.02)	1.73 (.02)	.98 (.03)	1.38 (.04)	2.07 (.05)	1.74 (.07)	a,b,c,d,e
**Marital status at *t*_1_, %**							
** Married/partnered**	40.3	54.4	52.1	51.9	61.4	50.6	a,b,c,d,e
** Divorced/separated**	26.9	15.2	18.8	22.4	15.1	26.1	a,b,c,d
** Widowed**	24.1	25.0	22.5	18.9	18.4	17.9	c,d,e
** Never married**	8.7	5.5	6.6	6.9	5.1	5.5	a,b,d
**Lived alone at t_1_, %**							
** No**	55.5	80.8	72.7	74.0	80.4	71.5	a,b,c,d,e
** Yes**	44.5	19.2	27.3	26.0	19.6	28.5	a,b,c,d,e
**Number of proximate children at *t*_1_, mean (*SD*)**	.78 (.03)	1.04 (.03)	1.00 (.03)	.94 (.04)	1.13 (.06)	.94 (.07)	a,b,c,d,e
**Spouse dead between *t*_1_ and *t*_2_, %**							
** No**	96.6	98.0	96.9	94.6	96.2	87.6	a,c,e
** Yes**	3.4	2.0	3.1	5.4	3.8	12.4	a,c,e
**Divorced/separated between *t*_1_ and *t*_2_, %**							
** No**	98.7	99.5	99.3	96.5	98.2	93.6	a,c,e
** Yes**	1.3	0.5	0.7	3.5	1.8	6.4	a,c,e
**Received formal care at *t*_1_, %**							
** No**	96.0	92.5	91.8	92.9	94.2	89.1	a,b,c,e
** Yes**	4.0	7.5	8.2	7.1	5.8	10.9	a,b,c,e
**Received formal care at *t*_2_, %**							
** No**	93.9	88.7	89.5	86.8	83.1	82.7	a,b,c,d,e
** Yes**	6.1	11.3	10.5	13.2	16.9	17.3	a,b,c,d,e
**Rural residence, %**							
** No**	74.2	73.4	72.1	75.0	76.7	78.1	
** Yes**	25.8	26.6	27.9	25.0	23.3	21.9	

*Note*. Data are from the 2010–2018 Waves of the Health and Retirement Study (*n *= 4,160 respondents; 8,332 person-year observations). The letters in the “*t*-tests” column indicate a significant difference in mean/proportion of the respective variable, between person-years with no informal care over time and those with: persistent care (a), care addition (b), total care loss (c), partial care loss (d), or care transition (e) at the .05 level. SD = standard deviation; ADL = activities of daily living; IADL = instrumental activities of daily living.

There were significant differences in other variables across care stability categories. Respondents without informal care over time reported the fewest depressive symptoms (*M* = 2.92), significantly lower than those of all other care stability categories except total care loss. Respondents without informal care over time also reported fewer ADL and IADL limitations, fewer depressive symptoms, and fewer chronic conditions at *t*_1_. Moreover, those who did not receive care were less likely to be married (40.3%), more likely to live alone (44.5%), and had fewer children living nearby (*M* = .78), relative to others. Conversely, a significantly higher proportion of respondents who experienced total care loss or care transition reported spousal death or divorce between *t*_1_ and *t*_2_, vis-à-vis those with no care receipt or persistent care (*p* < .05).

**Figure 2.**
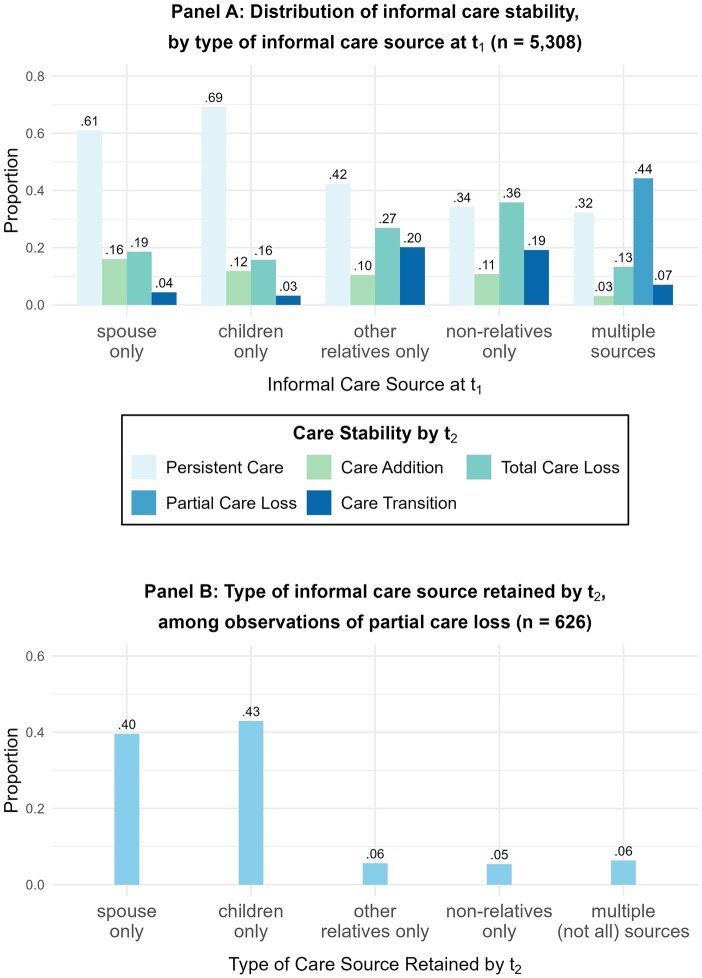
Patterns of informal care stability by informal caregiver type. Panel A presents the distribution of informal care stability by type of informal care source at *t*_1_ (*n* = 5,308). The numbers above bars indicate the relative proportion within each type of *t*_1_ informal care source. Observations with no informal care receipt at *t*_1_ are not included. Panel B presents the type of informal care source retained by *t*_2_ among observations of partial care loss (*n* = 626). The numbers above bars indicate proportions within this sample.

Experiences of care instability also varied by caregiver type. Figure 2, Panel A shows that among older adults receiving care from spouses only or children only at *t*_1_, most experienced persistent care (61% and 69%, respectively), although care transition was rare. Even so, a nonnegligible proportion of these respondents reported losing care from spouses (19%) or children (16%) without replacement caregivers. By comparison, experiences of care transition and total care loss were more common among respondents with other-relative or nonrelative caregivers at *t*_1_. Panel B shows that among respondents who experienced partial care loss, spouses (40%) and children (43%) were the most commonly retained caregivers.

**Figure 3.**
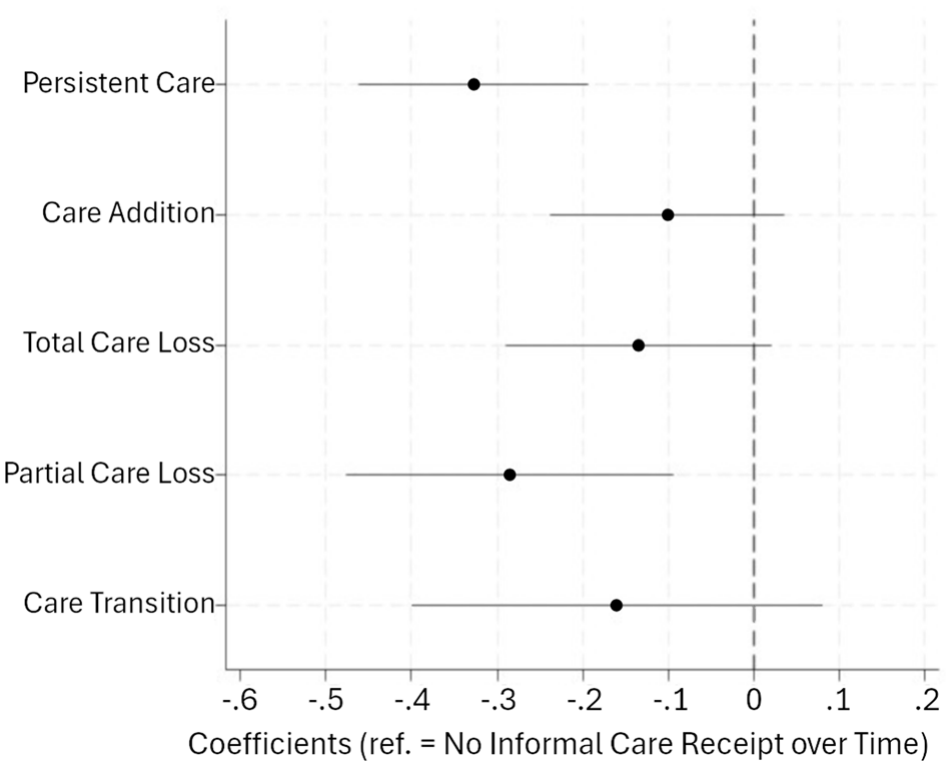
Coefficients of the informal care stability measure, from the mixed-effect model predicting depressive symptoms at *t*_2_ (*n* = 8,332). Results are based on Model 1.1, Supplementary Table 1 (see online supplementary material). Compared to persistent care, care addition and total care loss categories are also associated with significantly greater depressive symptoms (*p* < .001 and .01, respectively).

Figure 3 presents the regression coefficients for informal care stability predicting depressive symptoms at *t*_2_, adjusting for covariates (based on Model 1.1, Supplementary Table 1, see online supplementary material). Individuals with persistent informal care saw significantly fewer depressive symptoms than those who did not receive informal care over time (*b* = −.351; *p* < .001). Partial care loss also predicted lower depression than those who reported no informal care at both timepoints (*b* = −.328; *p* < .01).

Conversely, care addition, total care loss, and care transition were not significantly associated with depressive symptoms. Though their coefficients were negative, the size of these effects was considerably smaller than those of persistent care and partial care loss. Indeed, relative to those with persistent care, respondents who experienced care addition or total care loss reported significantly greater depressive symptoms (*b* = .220 and .207; *p* < .001 and .01, respectively).

In terms of the moderating effects of informal care stability, we found no significant interaction between care stability and t_2_ ADL limitations (Model 1.2, Supplementary Table 1, see online supplementary material). By contrast, persistent care, care addition, and partial care loss (vs. no informal care over time) significantly attenuated the association between *t*_2_ IADL limitations and depressive symptoms. Model 1.3 suggests that among respondents with no informal care over time, each additional IADL limitation was associated with a .382 increase in depressive symptoms. However, this association was smaller for respondents with persistent care (.229), care addition (.180), and partial care loss (.161).

**Figure 4.**
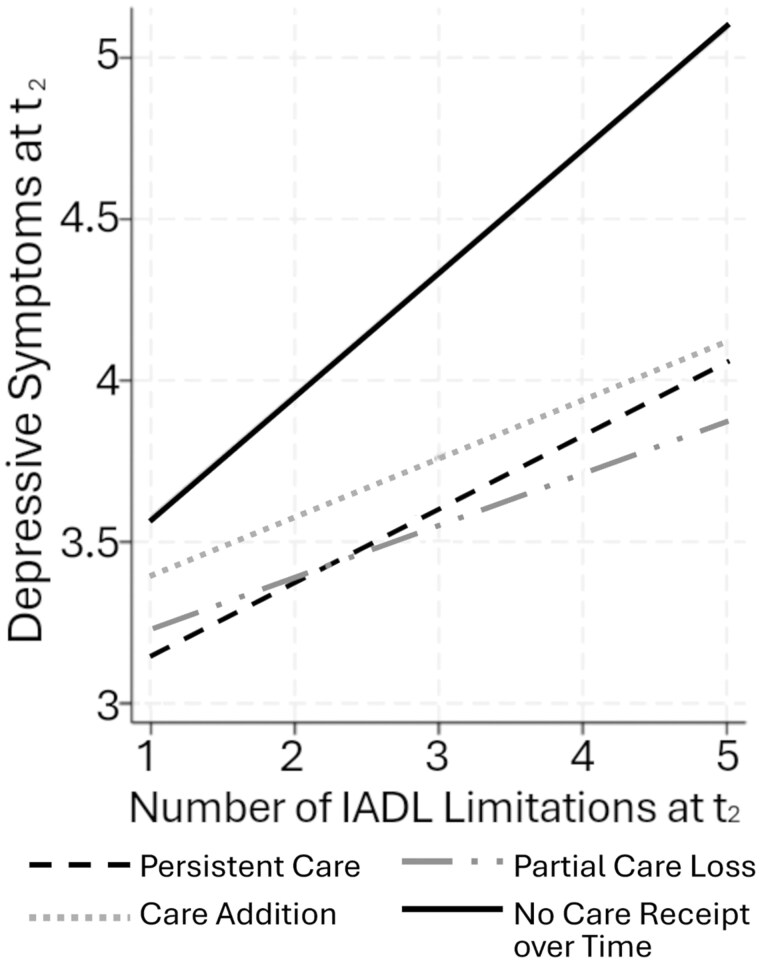
Predicted values of depressive symptoms at *t*_2_, by number of instrumental activities of daily living (IADL) limitations at *t*_2_ and informal care stability. Results are based on Model 1.3, Supplementary Table 1 (see online supplementary material).

Figure 4 illustrates these moderation patterns. Among respondents with few IADL limitations, the predicted values of *t*_2_ depressive symptoms did not substantially differ across care stability status. However, mental health gaps were considerably widened for those with more IADL limitations. For instance, among respondents with four IADL limitations, the predicted value of *t*_2_ depressive symptoms is 4.71 for those without informal care, around 20%-25% higher than those with persistent care (3.84), care addition (3.94), or partial care loss (3.72).

### Supplemental analysis

We examined whether the mental health implications of informal care stability varied by *t*_1_ caregiver type, and whether the implications from partial care loss varied by the type of source retained. See *Supplementary Material*  Section I: Supplementary Method for full descriptions of these results (see online supplementary material). In terms of variations by the initial caregiver type, results are generally consistent with the main analysis (Supplementary Figure 1, see online supplementary material; full regression results are reported in Supplementary Table 2, see online supplementary material). The main difference is that care transition was indeed associated with better mental health among older adults with other-relative or nonrelative caregivers only at *t*_1,_ but not among those receiving care from spouses only or children only at *t*_1_. As for variations within partial care loss, its apparent mental health benefits were pronounced only for those who retained either spousal or child care, not for those who retained care from other relatives or non-relatives (Supplementary Figure 2, see online supplementary material; full regression results are reported in Supplementary Table 3, see online supplementary material). Together, these results reveal that informal care stability is particularly important for the psychological well-being of adults receiving care from close family members.

We also investigated the heterogeneity in the association between informal care stability and depressive symptoms by individuals’ limitation progressions, measured through changes in the numbers of ADL and IADL limitations (*t*_2_-*t*_1_). Results suggest that persistent care and partial care loss still predict lower depression after controlling for limitation progressions (Supplementary Table 4, see online supplementary material). Meanwhile, the two limitation progressions did not moderate the care stability–depressive symptoms associations, indicating no heterogeneity.

We examined potential heterogeneity by formal care receipt. We found that *t*_1_ formal care receipt did not moderate the care stability–depression associations (Model 5.1, Supplementary Table 5, see online supplementary material). However, the interaction between receiving formal care at *t*_2_ and informal care transition significantly predicted *t*_2_ depressive symptoms (Model 5.2). Supplementary Figure 3 (see online supplementary material) illustrates that among respondents without formal care at *t*_2_, depressive symptoms did not vary between care transition and no informal care. Among those with formal care at *t*_2_, care transition predicted significantly lower depressive symptoms (2.90) than no informal care (3.85; *p* < .01). The implications are discussed in the next section.

In addition, we ran regressions with balanced inverse probability weights that account for attrition. Results are similar to the main findings (Supplementary Table 6, see online supplementary material).

## Discussion

Little research has examined how the longitudinal patterns of informal care shape recipients’ mental health. This study investigates how the receipt of stable and unstable informal care predicts depressive symptoms, and how informal care stability attenuates the likely harmful mental health effects of functional limitations among older adults with persistent care needs.

The results reveal that respondents with persistent care over time, as well as those who experienced partial care loss, reported fewer depressive symptoms than individuals without informal care over time. By contrast, respondents who experienced care addition, total care loss, or care transition did not see a similar mental health benefit. Those who experienced care addition or total care loss also reported more depressive symptoms than those receiving persistent care. In additional analysis, we found that the mental health benefits of persistent care and partial care loss were primarily observed among older adults who retained care from close family members, i.e., spouses and children.

These findings highlight the importance of stable informal care from close family members for the mental health of older adults with persistent (I)ADL limitations. Scholars have posited that the stability of informal care facilitates a strong relationship between caregivers and care recipients, which may enhance care quality (Allen et al., 2012). This study advances these implications by demonstrating that retaining care from close family members is critical for care recipients’ mental health. Close family caregivers tend to better understand older adults’ preferences (Floridi et al., 2024; Swinkels et al., 2025) and provide assistance with a broader range of daily tasks, especially those related to ADL limitations (Allen et al., 2012). Their persistent care may sustain high-quality support and promote older adults’ social integration, by reinforcing connections with important others and mitigating feelings of abandonment (Floridi et al., 2024). In contrast, the loss of close family caregivers—even if replaced by an extended family or non-kin caregiver—may result in reduced support over daily activities, thereby elevating the risk of unmet care needs among older adults.

Hence, informal care stability may serve as a measure of care quality from a longitudinal perspective, especially for older adults with close family caregivers. This has important implications for assessing informal care delivery over time, as most older adults draw support primarily from their closest kin.

Our paper also makes an important methodological contribution. Existing observational studies have primarily analyzed how point-in-time measures of informal care receipt predict mental health, often finding a negative association between the two (Coe et al., 2021; Kwak et al., 2014; Shang & Patterson, 2024). Our analyses indicate that the mental health benefits of informal care are more pronounced among respondents with persistent care needs. By explicitly capturing within-person changes in care receipt, our research underscores the importance of considering the temporal dynamics of informal care to fully understand its well-being consequences.

Interestingly, we found weak evidence of a direct link between adding caregivers and depressive symptoms. One possible explanation is that having more caregivers increases care recipients’ feelings of self-burden to others, thereby introducing negative self-perceptions of aging (Kwak et al., 2014). Care addition may also be a result of increased care needs due to worsening health conditions, which deteriorate mental health.

We also observed that stable informal care weakens the negative relationship between IADL limitations and depressive symptoms. Specifically, experiences of persistent care, care addition, and partial care loss—representing more stable informal care receipt—attenuated the association between the number of IADL limitations and depressive symptoms. These findings highlight the importance of delivering high-quality care to meet the needs of more individual-specific, instrumental activities in older adults’ daily routines. The specific tasks comprised in IADLs vary by personal preferences and social contexts, and supporting older adults in these activities may require caregivers to develop strong relationships with them (Bruderer-Hofstetter et al., 2022; Nygård, 2003). A stable source of informal care may contribute to a better understanding of recipients’ needs and, thus, the delivery of high-quality care (Allen et al., 2012).

By comparison, informal care stability does not moderate the harmful mental health effects of ADL limitations. Scholars have recognized that ADL limitations are more serious forms of disabilities, compared to IADL limitations (Spector et al., 1987). The resultant psychological distress from health deterioration, coupled with negative feelings such as diminished self-esteem and loneliness, may be hard to buffer through informal care (Na & Streim, 2017; Xiao et al., 2022).

In *supplemental analyses*, we found that the association between informal care stability and mental health did not vary by limitation progressions, implying an independent role played by care stability in shaping psychological well-being. However, we found that receiving formal care contributed to a mental health benefit associated with care transition. One potential explanation is that the additional receipt of formal care could be the result of coordination across care sources, which may improve care quality. The combined use of formal and informal care may also yield mental health benefits, for example, by enhancing perceived mastery among older adults (Wylie et al., 2023).

The study has several limitations. First, the analyses only examined associations and could not make causal claims. Prior studies have stressed that observational studies of informal care receipt and health may be endogenous (Coe et al., 2021; Pristavec & Luth, 2020). However, the caveats mainly concerned findings of negative associations between informal care receipt and health. In contrast, this study found a mental health benefit from receiving stable informal care, partially owing to a wide range of control factors.

Second, due to data limitations, the analyses could not observe potential care transitions within children, other relatives, and nonrelatives. It is possible, for instance, that turnover occurred between different child caregivers and influenced recipients’ mental health. Scholars have suggested that transitions between caregivers across different domains of relationships may be the primary indicator of care quality, due to their different implications of care propensity and proximity to the recipient (Allen et al., 2012). By extension, this study finds that care instability across relationship domains has important mental health consequences. Additionally, as these unobserved changes were most likely classified as persistent care in our analyses, they might attenuate the estimated effects of care instability and yield conservative estimates.

Third, the study could only observe informal care stability over 2-year intervals. It is likely that some respondents who were classified as receiving persistent care indeed experienced care loss/transitions within this period. To the extent that care loss and transitions dampen the mental health benefits of informal care receipt, the analysis yielded conservative estimates of the care stability–depressive symptom associations. Additionally, while examining 2-year informal care changes offer insight into the short-term mental health impacts, future studies should examine longer-term trajectories for a more comprehensive assessment.

In conclusion, the study addresses an important gap in the research on informal care, by investigating the temporal dynamics of informal care receipt and its well-being implications. Our findings have important implications for long-term care policies, which have long focused on the provision of sustainable care to individuals with persistent needs (Allen et al., 2012). Though close family members may feel obligated by social norms to provide informal care, financial incentives (e.g., tax deductions, caregiving allowances) and the introduction or expansion of family leave programs may effectively relieve their burdens and encourage their retention. Our results suggest that such interventions could sustain high-quality care from close family members and help mitigate the psychological distress suffered by care recipients because of their long-term care needs. Another potential area of intervention concerns enhancing family caregivers’ support networks and resources. For instance, providing access to training, mental health resources, and respite care for caregivers can reduce burnout and improve their capacity to deliver stable care over time.

This is not to diminish the value of care provided by extended family members and non-relatives. Our findings show that older adults with these caregivers also reported fewer depressive symptoms when they experienced either persistent care or care transitions, whereas those experiencing total care loss did not see any mental health benefits. As extended family and non-kin caregivers might also need to support their own close relatives, policies should prioritize coordination among these caregivers. Care coordination efforts should include identifying suitable replacement caregivers when current ones need to transition out of the roles; they should also facilitate communication between outgoing and incoming caregivers regarding essential tasks, personal preferences, and medical information of the care recipient (Tang et al., 2018). These practices may sustain long-term care for older adults without immediate family support, promote their mental well-being, and mitigate caregiver burdens in the long run.

Future research should examine the relationship between informal care stability and broader dimensions of care quality, such as emotional support and autonomy, while also exploring the interplay between informal and formal care arrangements. By examining how these dynamics evolve and impact older adults’ well-being, new research can provide critical insights into designing sustainable and effective long-term care strategies for aging populations.

## Supplementary Material

gbaf154_Supplementary_Data

## Data Availability

The data used in the present study are publicly available on the Gateway to Global Aging Data website and can be accessed through registration at https://g2aging.org/. The analytic methods and codes for the statistical analysis are available upon request from the corresponding author. The study is not preregistered.
