# Supplementary material for: The mental health implications of informal care receipt stability among older adults with persistent care needs
Source: J Gerontol B Psychol Sci Soc Sci. 2025 Aug 21;80(11):gbaf154. doi: 10.1093/geronb/gbaf154 (PMC12517747; doi:10.1093/geronb/gbaf154)
Supplement: gbaf154_Supplementary_Data [file gbaf154_supplementary_data.zip › JGSS suppl Li, Angrisani, & Lee.docx]

***The Journals of Gerontology, Series B: Psychological Sciences and Social Sciences* Supplementary Material: Li, Angrisani, & Lee. The Mental Health Implications of Informal Care Receipt Stability among Older Adults with Persistent Care Needs.**

**I. Supplementary Method**

Analysis of Heterogeneity by Caregiver Types

We analyzed how the association between informal care instability and depressive symptoms varies by caregiver type at t_1_. The coefficients of care stability are presented in Figure S1 (see the Supplemental Figures section; Table S2 in the Supplemental Tables section reports the full regression). Most of these results are consistent with the main findings. First, persistent care across t_1_ caregiver type was generally associated with significantly lower depressive symptoms, compared to no informal care receipt over time. Its coefficient is -.377 for observations receiving care from spouses only at t_1_ (*p* < .001), -.345 for observations receiving care from children only at t_1_ (*p* < .001), -.667 for observations receiving care from other relatives only at t_1_ (*p* < .01), and -.308 for observations receiving care from multiple sources at t_1_ (*p* < .01). The only exception was persistent care from non-relatives at t_1_, which was marginally associated with fewer depressive symptoms due to its smaller cell count (*p* = .055); the magnitude of its coefficient (-.415) is on par with persistent care from other sources.

Second, we found weak evidence suggesting a mental health benefit associated with care addition, as most of its coefficients were not significant at .05 level. The exceptions are those receiving care from spouses only at t_1_, for whom care addition predicted better mental health (*b* = -.308; *p* < .01). One potential explanation is that having additional caregivers reduced the caregiving burden on spouses, whose lower stress may improve the mental health of their partners, i.e., the care recipient, through social contagion of mental health within households.

Third, total care loss was not associated with depressive symptoms at all, compared to no informal care receipt over time.

Fourth, and most importantly, we observed considerable heterogeneity in care transition by initial care arrangement. For individuals with spousal or child caregivers only at t_1_, care transition was not significantly associated with depressive symptoms (indeed, their coefficients were positive). However, care transition predicted significantly better mental health for individuals with other-relative caregivers at t_1_ (*b* = -1.066; *p* < .001) or non-relative caregivers at t_1_ (*b* = -.564; *p* < .05), compared to those without informal care at both time points.

We then analyzed the heterogeneity in partial care loss experienced by individuals with multiple care sources at t_1_, by the type of care source they retained at t_2_. Results are presented in Figure S2 (Table S3 presents full regression). We found that partial care loss was associated with significantly better mental health only for those who retained care from close family members, i.e., spouses (*b* = -.513; *p* < .001) and children (*b* = -.328; *p* < .05). It was not associated with depressive symptoms for those who retained care only from other relatives or non-relatives.

**II. Supplementary Figures**

**Supplementary Figure 1.** Coefficients of the informal care stability by caregiver type at t_1_, from the mixed-effect model predicting depressive symptoms at t_2_ (n = 8,332). Results are based on Model 2, Table S2. The model also controls for number of ADL limitations at t_2_, number of IADL limitations at t_2_, and covariates specified in the Measures section. In the interest of space, the graph does not display the coefficients of care addition for observations with no informal care receipt at t_1_ (not significant at the .05 level) and partial care loss (similar result to the main analysis; disaggregation by type of source retained was analyzed and presented in Figure S2).

**
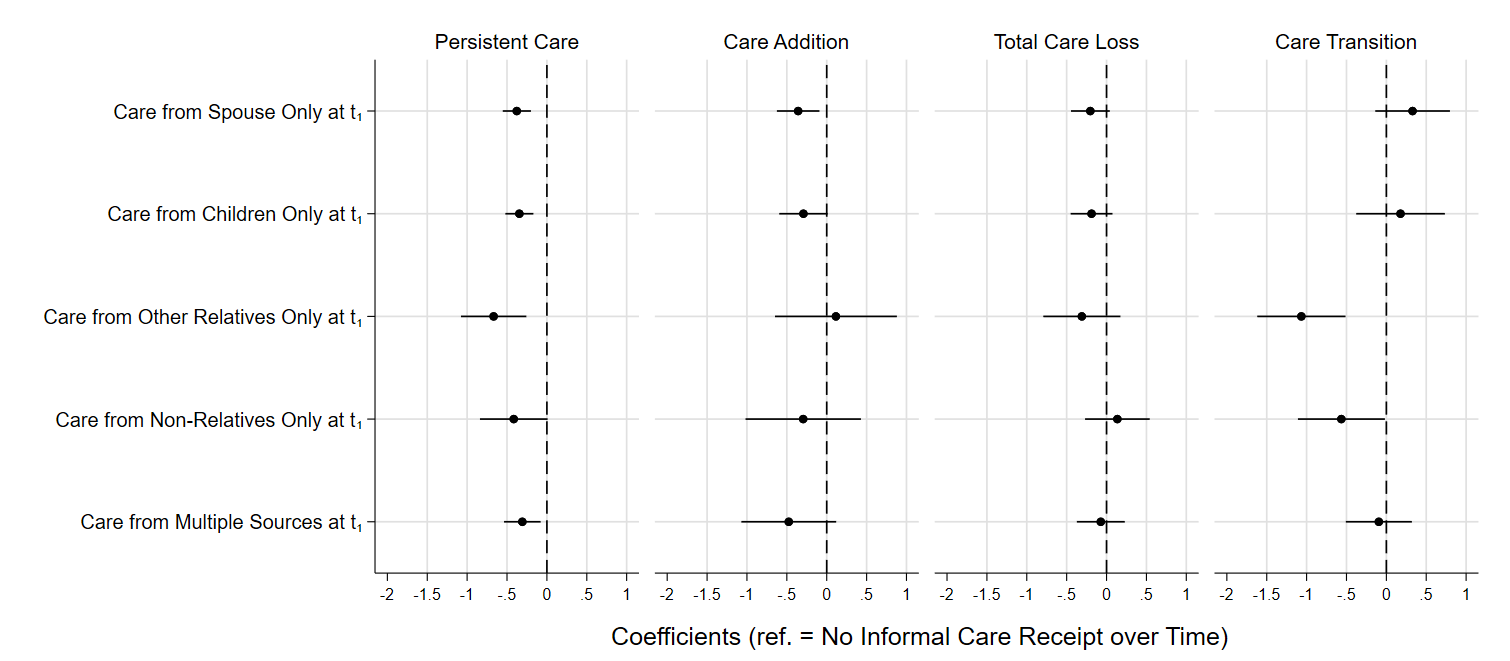
**

**Supplementary Figure 2.** Coefficients of the partial care loss by type of informal caregiver retained at t_2_, from the mixed-effect model predicting depressive symptoms at t_2_ (n = 8,332). Results are based on Model 3, Table S3. The model also controls for other informal care stability categories, number of ADL limitations at t_2_, number of IADL limitations at t_2_, and covariates specified in the Measures section.

**
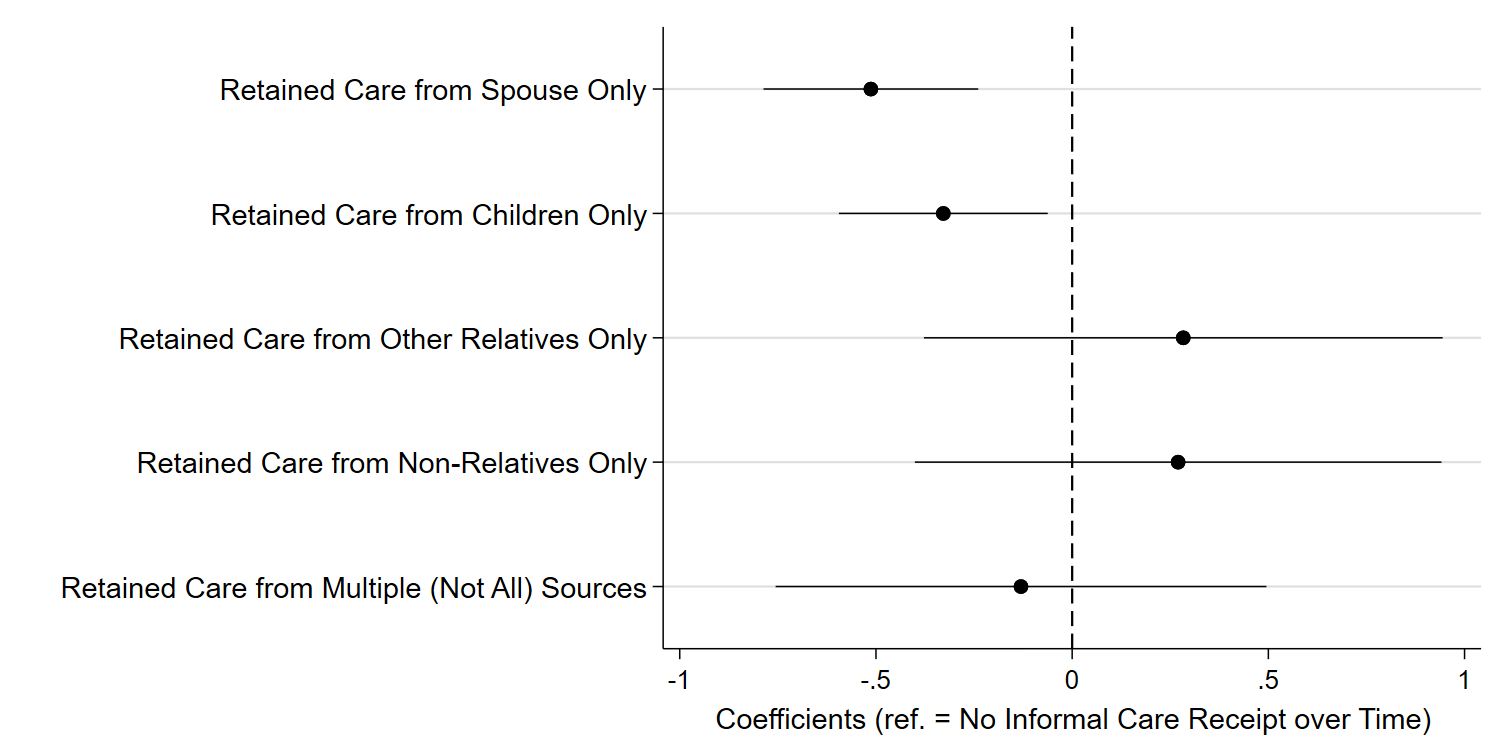
**

**Supplementary Figure 3.** Predicted depressive symptoms at t_2_, by formal care receipt at t_2_ and informal care stability (care transition vs. no informal care receipt over time; n = 8,332). Results are based on Model 5.2, Table S5.

**
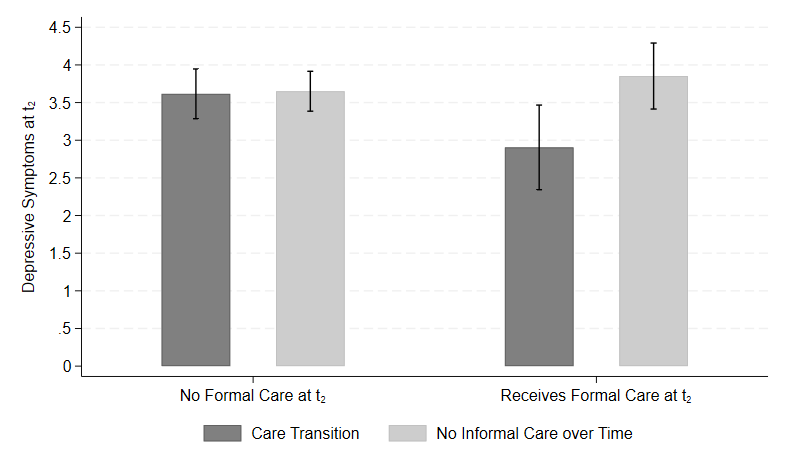
**

**III. Supplementary Tables**

**Supplementary Table 1.** Mixed-effect regressions predicting depressive symptoms at t_2_ (n = 8,332).

|  | **Model 1.1** | **Model 1.2** | **Model 1.3** |
| --- | --- | --- | --- |
|  | Direct Effects of Care Stability | Interactions with ADL Limitations | Interactions with IADL Limitations |
| Informal Care Instability (ref. = No Informal Care Receipt over Time) | |  |  |
| Persistent Care | -0.345*** | -0.305** | -0.267** |
|  | (0.07) | (0.10) | (0.09) |
| Care Addition | -0.125 | -0.011 | 0.037 |
|  | (0.07) | (0.11) | (0.10) |
| Total Care Loss | -0.138 | -0.227 | -0.128 |
|  | (0.08) | (0.12) | (0.10) |
| Partial Care Loss | -0.320** | -0.388* | -0.115 |
|  | (0.10) | (0.15) | (0.15) |
| Care Transition | -0.166 | 0.123 | -0.009 |
|  | (0.12) | (0.20) | (0.21) |
| No. of ADL Limitations at t_2_ (ADL) | 0.195*** | 0.221*** | 0.199*** |
|  | (0.02) | (0.04) | (0.02) |
| ADL * Persistent Care |  | -0.028 |  |
|  |  | (0.04) |  |
| ADL * Care Addition |  | -0.060 |  |
|  |  | (0.05) |  |
| ADL * Total Care Loss |  | 0.053 |  |
|  |  | (0.06) |  |
| ADL * Partial Care Loss |  | 0.015 |  |
|  |  | (0.06) |  |
| ADL * Care Transition |  | -0.134 |  |
|  |  | (0.07) |  |
| No. of IADL Limitations at t_2_ (IADL) | 0.232*** | 0.235*** | 0.382*** |
|  | (0.02) | (0.02) | (0.06) |
| IADL * Persistent Care |  |  | -0.153* |
|  |  |  | (0.07) |
| IADL * Care Addition |  |  | -0.202** |
|  |  |  | (0.07) |
| IADL * Total Care Loss |  |  | -0.049 |
|  |  |  | (0.09) |
| IADL * Partial Care Loss |  |  | -0.221** |
|  |  |  | (0.08) |
| IADL * Care Transition |  |  | -0.191 |
|  |  |  | (0.10) |
| Age | -0.096*** | -0.095*** | -0.095*** |
|  | (0.03) | (0.03) | (0.03) |
| Age Squared | 0.000* | 0.000* | 0.000* |
|  | (0.00) | (0.00) | (0.00) |
| Female | 0.177*** | 0.180*** | 0.180*** |
|  | (0.05) | (0.05) | (0.05) |
| Race (ref. = White) |  |  |  |
| Black | -0.148* | -0.152* | -0.150* |
|  | (0.06) | (0.06) | (0.06) |
| Latino | 0.125 | 0.122 | 0.120 |
|  | (0.07) | (0.07) | (0.07) |
| Other | -0.022 | -0.033 | -0.029 |
|  | (0.13) | (0.13) | (0.13) |
| Educational Attainment (ref. = Less than High School) |  |  |  |
| High School Graduate | -0.056 | -0.057 | -0.061 |
|  | (0.06) | (0.06) | (0.06) |
| Some College | -0.163* | -0.163* | -0.165* |
|  | (0.07) | (0.07) | (0.07) |
| BA Degree | -0.336*** | -0.336*** | -0.341*** |
|  | (0.09) | (0.09) | (0.09) |
| Logged Household Wealth at t_1_ | -0.438 | -0.453 | -0.445 |
|  | (0.28) | (0.28) | (0.28) |
| CES-D at t_1_ (ref. = 0-1) |  |  |  |
| 2-4 | 1.070*** | 1.066*** | 1.066*** |
|  | (0.05) | (0.05) | (0.05) |
| 5-8 | 2.647*** | 2.641*** | 2.648*** |
|  | (0.06) | (0.06) | (0.06) |
| Missing due to Proxy Interview | 0.916*** | 0.912*** | 0.921*** |
|  | (0.19) | (0.19) | (0.19) |
| No. of Chronic Disease at t_1_ | 0.093*** | 0.093*** | 0.093*** |
|  | (0.02) | (0.02) | (0.02) |
| No. of ADL Limitations at t_1_ | -0.044* | -0.045** | -0.043* |
|  | (0.02) | (0.02) | (0.02) |
| No. of IADL Limitations at t_1_ | -0.003 | -0.002 | -0.003 |
|  | (0.02) | (0.02) | (0.02) |
| Marital Status at t_1_ (ref. = Married/Partnered) |  |  |  |
| Divorced/Separated | 0.176* | 0.170* | 0.168* |
|  | (0.07) | (0.07) | (0.07) |
| Widowed | 0.029 | 0.027 | 0.026 |
|  | (0.08) | (0.08) | (0.08) |
| Never Married | 0.009 | 0.007 | 0.009 |
|  | (0.11) | (0.11) | (0.11) |
| Number of Proximate Children at t_1_ | -0.026 | -0.026 | -0.025 |
|  | (0.02) | (0.02) | (0.02) |
| Spouse Dead between t_1_ and t_2_ | 0.721*** | 0.711*** | 0.711*** |
|  | (0.12) | (0.12) | (0.12) |
| Divorced/Separated between t_1_ and t_2_ | 0.160 | 0.142 | 0.139 |
|  | (0.19) | (0.19) | (0.19) |
| Lived Alone at t_1_ | 0.010 | 0.006 | -0.000 |
|  | (0.07) | (0.07) | (0.07) |
| Received Formal Care at t_1_ | -0.071 | -0.067 | -0.082 |
|  | (0.10) | (0.10) | (0.10) |
| Received Formal Care at t_2_ | -0.063 | -0.073 | -0.075 |
|  | (0.08) | (0.08) | (0.08) |
| Rural Residence | 0.005 | 0.001 | 0.000 |
|  | (0.06) | (0.06) | (0.06) |
| Year Dummies (ref. = 2012) |  |  |  |
| 2014 | -0.072 | -0.071 | -0.076 |
|  | (0.06) | (0.06) | (0.06) |
| 2016 | -0.177** | -0.176** | -0.181** |
|  | (0.06) | (0.06) | (0.06) |
| 2018 | -0.152* | -0.151* | -0.158** |
|  | (0.06) | (0.06) | (0.06) |
| Constant | 12.547** | 12.714** | 12.535** |
|  | (4.30) | (4.30) | (4.30) |
| **N** | **8332** | **8332** | **8332** |

* p<.05, ** p<.01, *** p<.001

**Supplementary Table 2.** Mixed-effect regression predicting depressive symptoms at t_2_ on informal care instability, disaggregated by caregiver type at t_1_ (n = 8,332).

|  | **Model 2** |
| --- | --- |
| Informal Care Instability (ref. = No Informal Care Receipt over Time) |  |
| Persistent Care, Only Spouse Care at t_1_ | -0.377*** |
|  | (0.09) |
| Care Addition, Only Spouse Care at t_1_ | -0.358** |
|  | (0.14) |
| Total Care Loss, Only Spouse Care at t_1_ | -0.203 |
|  | (0.12) |
| Care Transition, Only Spouse Care at t_1_ | 0.329 |
|  | (0.24) |
| Persistent Care, Only Children Care at t_1_ | -0.345*** |
|  | (0.09) |
| Care Addition, Only Children Care at t_1_ | -0.292 |
|  | (0.15) |
| Total Care Loss, Only Children Care at t_1_ | -0.188 |
|  | (0.13) |
| Care Transition, Only Children Care at t_1_ | 0.178 |
|  | (0.28) |
| Persistent Care, Only Other Relatives Care at t_1_ | -0.667** |
|  | (0.21) |
| Care Addition, Only Other Relatives Care at t_1_ | 0.115 |
|  | (0.39) |
| Total Care Loss, Only Other Relatives Care at t_1_ | -0.309 |
|  | (0.25) |
| Care Transition, Only Other Relatives Care at t_1_ | -1.066*** |
|  | (0.28) |
| Persistent Care, Only Non-Relatives Care at t_1_ | -0.415 |
|  | (0.22) |
| Care Addition, Only Non-Relatives Care at t_1_ | -0.295 |
|  | (0.37) |
| Total Care Loss, Only Non-Relatives Care at t_1_ | 0.136 |
|  | (0.21) |
| Care Transition, Only Non-Relatives Care at t_1_ | -0.564* |
|  | (0.28) |
| Persistent Care, Multiple Care Sources at t_1_ | -0.308** |
|  | (0.12) |
| Care Addition, Multiple Care Sources at t_1_ | -0.475 |
|  | (0.30) |
| Total Care Loss, Multiple Care Sources at t_1_ | -0.072 |
|  | (0.15) |
| Partial Care Loss, Multiple Care Sources at t_1_ | -0.347*** |
|  | (0.10) |
| Care Transition, Multiple Care Sources at t_1_ | -0.094 |
|  | (0.21) |
| Care Addition, No Informal Care Receipt at t_1_ | -0.036 |
|  | (0.08) |
| No. of ADL Limitations at t_2_ | 0.232*** |
|  | (0.02) |
| No. of IADL Limitations at t_2_ | 0.196*** |
|  | (0.02) |
| No. of ADL Limitations at t_1_ | -0.044** |
|  | (0.02) |
| No. of IADL Limitations at t_1_ | 0.011 |
|  | (0.02) |
| CES-D at t_1_ (ref. = 0-1) |  |
| 2-4 | 1.065*** |
|  | (0.05) |
| 5-8 | 2.634*** |
|  | (0.06) |
| Missing due to Proxy Interview | 0.913*** |
|  | (0.18) |
| No. of Chronic Disease at t_1_ | 0.094*** |
|  | (0.02) |
| Age | -0.094*** |
|  | (0.03) |
| Age Squared | 0.000* |
|  | (0.00) |
| Female | 0.173*** |
|  | (0.05) |
| Race (ref. = White) |  |
| Black | -0.145* |
|  | (0.06) |
| Latino | 0.114 |
|  | (0.07) |
| Other | -0.036 |
|  | (0.13) |
| Educational Attainment (ref. = Less than High School) |  |
| High School Graduate | -0.061 |
|  | (0.06) |
| Some College | -0.167* |
|  | (0.07) |
| BA Degree | -0.336*** |
|  | (0.09) |
| Marital Status at t1 (ref. = Married/Partnered) |  |
| Divorced/Separated | 0.180* |
|  | (0.08) |
| Widowed | 0.023 |
|  | (0.09) |
| Never Married | 0.020 |
|  | (0.11) |
| Number of Proximate Children at t_1_ | -0.029 |
|  | (0.02) |
| Lived Alone at t_1_ | -0.002 |
|  | (0.07) |
| Spouse Dead between t_1_ and t_2_ | 0.657*** |
|  | (0.12) |
| Divorced/Separated between t_1_ and t_2_ | 0.077 |
|  | (0.19) |
| Received Formal Care at t_1_ | -0.066 |
|  | (0.10) |
| Received Formal Care at t_2_ | -0.073 |
|  | (0.08) |
| Logged Household Wealth at t_1_ | -0.438 |
|  | (0.28) |
| Rural Residence | 0.007 |
|  | (0.06) |
| Year Dummies (ref. = 2012) |  |
| 2014 | -0.077 |
|  | (0.06) |
| 2016 | -0.182** |
|  | (0.06) |
| 2018 | -0.158** |
|  | (0.06) |
| Constant | 12.523** |
|  | (4.32) |
| * p<.05, ** p<.01, *** p<.001 |  |

**Supplementary Table 3.** Mixed-effect regression predicting depressive symptoms at t_2_ on informal care instability, with partial care loss disaggregated by type of caregiver retained at t_2_ (n = 8,332).

|  | **Model 3** |
| --- | --- |
| Informal Care Instability (ref. = No Informal Care Receipt over Time) |  |
| Persistent Care | -0.348*** |
|  | (0.07) |
| Care Addition | -0.126 |
|  | (0.07) |
| Total Care Loss | -0.139 |
|  | (0.08) |
| Partial Care Loss, Retained Care from Spouse Only | -0.513*** |
|  | (0.14) |
| Partial Care Loss, Retained Care from Children Only | -0.328* |
|  | (0.14) |
| Partial Care Loss, Retained Care from Other Relatives Only | 0.283 |
|  | (0.34) |
| Partial Care Loss, Retained Care from Other Non-Relatives Only | 0.270 |
|  | (0.34) |
| Partial Care Loss, Retained Care from Multiple (But not All) Sources | -0.130 |
|  | (0.32) |
| Care Transition | -0.164 |
|  | (0.12) |
| No. of ADL Limitations at t_2_ | 0.194*** |
|  | (0.02) |
| No. of IADL Limitations at t_2_ | 0.232*** |
|  | (0.02) |
| CES-D at t1 (ref. = 0-1) |  |
| 2-4 | 1.072*** |
|  | (0.05) |
| 5-8 | 2.653*** |
|  | (0.06) |
| Missing due to Proxy Interview | 0.919*** |
|  | (0.18) |
| No. of Chronic Disease at t_1_ | 0.094*** |
|  | (0.02) |
| No. of ADL Limitations at t_1_ | -0.045** |
|  | (0.02) |
| No. of IADL Limitations at t_1_ | -0.003 |
|  | (0.02) |
| Age | -0.096*** |
|  | (0.03) |
| Age Squared | 0.000* |
|  | (0.00) |
| Female | 0.175*** |
|  | (0.05) |
| Race (ref. = White) |  |
| Black | -0.150* |
|  | (0.06) |
| Latino | 0.128 |
|  | (0.07) |
| Other | -0.019 |
|  | (0.13) |
| Educational Attainment (ref. = Less than High School) |  |
| High School Graduate | -0.059 |
|  | (0.06) |
| Some College | -0.166* |
|  | (0.07) |
| BA Degree | -0.339*** |
|  | (0.09) |
| Marital Status at t_1_ (ref. = Married/Partnered) |  |
| Divorced/Separated | 0.161* |
|  | (0.07) |
| Widowed | 0.014 |
|  | (0.08) |
| Never Married | -0.009 |
|  | (0.11) |
| Number of Proximate Children at t_1_ | -0.029 |
|  | (0.02) |
| Lived Alone at t_1_ | 0.004 |
|  | (0.07) |
| Spouse Dead between t_1_ and t_2_ | 0.710*** |
|  | (0.12) |
| Divorced/Separated between t_1_ and t_2_ | 0.140 |
|  | (0.19) |
| Received Formal Care at t_1_ | -0.070 |
|  | (0.10) |
| Received Formal Care at t_2_ | -0.064 |
|  | (0.08) |
| Logged Household Wealth at t_1_ | -0.445 |
|  | (0.28) |
| Rural Residence | 0.007 |
|  | (0.06) |
| Year Dummies (ref. = 2012) |  |
| 2014 | -0.071 |
|  | (0.06) |
| 2016 | -0.175** |
|  | (0.06) |
| 2018 | -0.149* |
|  | (0.06) |
| Constant | 12.664** |
|  | (4.30) |
| * p<.05, ** p<.01, *** p<.001 |  |

**Supplementary Table 4.** Mixed-effect regressions predicting depressive symptoms at t_2_, with progressions in (I)ADL limitations as independent variables (n = 8,332).

|  | **Model 4.1** | **Model 4.2** | **Model 4.3** |
| --- | --- | --- | --- |
|  | Direct Effects of Care Stability | Interactions with ADL Limitation Progressions | Interactions with IADL Limitation Progressions |
| Informal Care Instability (ref. = No Informal Care Receipt over Time) | |  |  |
| Persistent Care | -0.345*** | -0.343*** | -0.346*** |
|  | (0.07) | (0.07) | (0.07) |
| Care Addition | -0.125 | -0.127 | -0.147 |
|  | (0.07) | (0.07) | (0.08) |
| Total Care Loss | -0.138 | -0.120 | -0.110 |
|  | (0.08) | (0.08) | (0.09) |
| Partial Care Loss | -0.320** | -0.324** | -0.348*** |
|  | (0.10) | (0.10) | (0.10) |
| Care Transition | -0.166 | -0.156 | -0.182 |
|  | (0.12) | (0.12) | (0.13) |
| Change in No. of ADL Limitations (CADL) | 0.195*** | 0.204*** | 0.195*** |
|  | (0.02) | (0.04) | (0.02) |
| CADL * Persistent Care |  | -0.023 |  |
|  |  | (0.04) |  |
| CADL * Care Addition |  | -0.005 |  |
|  |  | (0.05) |  |
| CADL * Total Care Loss |  | 0.068 |  |
|  |  | (0.06) |  |
| CADL * Partial Care Loss |  | -0.043 |  |
|  |  | (0.06) |  |
| CADL * Care Transition |  | -0.063 |  |
|  |  | (0.07) |  |
| Change in No. of IADL Limitations (CIADL) | 0.232*** | 0.234*** | 0.246*** |
|  | (0.02) | (0.02) | (0.06) |
| CIADL * Persistent Care |  |  | -0.015 |
|  |  |  | (0.06) |
| CIADL * Care Addition |  |  | 0.017 |
|  |  |  | (0.07) |
| CIADL * Total Care Loss |  |  | 0.027 |
|  |  |  | (0.08) |
| CIADL * Partial Care Loss |  |  | -0.156 |
|  |  |  | (0.08) |
| CIADL * Care Transition |  |  | 0.054 |
|  |  |  | (0.10) |
| Age | -0.096*** | -0.095*** | -0.095*** |
|  | (0.03) | (0.03) | (0.03) |
| Age Squared | 0.000* | 0.000* | 0.000* |
|  | (0.00) | (0.00) | (0.00) |
| Female | 0.177*** | 0.178*** | 0.177*** |
|  | (0.05) | (0.05) | (0.05) |
| Race (ref. = White) |  |  |  |
| Black | -0.148* | -0.146* | -0.146* |
|  | (0.06) | (0.06) | (0.06) |
| Latino | 0.125 | 0.126 | 0.125 |
|  | (0.07) | (0.07) | (0.07) |
| Other | -0.022 | -0.028 | -0.021 |
|  | (0.13) | (0.13) | (0.13) |
| Educational Attainment (ref. = Less than High School) | |  |  |
| High School Graduate | -0.056 | -0.052 | -0.056 |
|  | (0.06) | (0.06) | (0.06) |
| Some College | -0.163* | -0.159* | -0.164* |
|  | (0.07) | (0.07) | (0.07) |
| BA Degree | -0.336*** | -0.334*** | -0.335*** |
|  | (0.09) | (0.09) | (0.09) |
| Logged Household Wealth at t_1_ | -0.438 | -0.442 | -0.451 |
|  | (0.28) | (0.28) | (0.28) |
| CES-D at t_1_ (ref. = 0-1) |  |  |  |
| 2-4 | 1.070*** | 1.068*** | 1.068*** |
|  | (0.05) | (0.05) | (0.05) |
| 5-8 | 2.647*** | 2.647*** | 2.644*** |
|  | (0.06) | (0.06) | (0.06) |
| Missing due to Proxy Interview | 0.916*** | 0.932*** | 0.892*** |
|  | (0.19) | (0.19) | (0.19) |
| No. of Chronic Disease at t_1_ | 0.093*** | 0.093*** | 0.094*** |
|  | (0.02) | (0.02) | (0.02) |
| No. of ADL Limitations at t_1_ | 0.230*** | 0.230*** | 0.231*** |
|  | (0.02) | (0.02) | (0.02) |
| No. of IADL Limitations at t_1_ | 0.151*** | 0.152*** | 0.150*** |
|  | (0.02) | (0.02) | (0.02) |
| Marital Status at t_1_ (ref. = Married/Partnered) |  |  |  |
| Divorced/Separated | 0.176* | 0.176* | 0.179* |
|  | (0.07) | (0.07) | (0.07) |
| Widowed | 0.029 | 0.030 | 0.028 |
|  | (0.08) | (0.08) | (0.08) |
| Never Married | 0.009 | 0.007 | 0.010 |
|  | (0.11) | (0.11) | (0.11) |
| Number of Proximate Children at t_1_ | -0.026 | -0.026 | -0.025 |
|  | (0.02) | (0.02) | (0.02) |
| Spouse Dead between t_1_ and t_2_ | 0.721*** | 0.724*** | 0.716*** |
|  | (0.12) | (0.12) | (0.12) |
| Divorced/Separated between t_1_ and t_2_ | 0.160 | 0.163 | 0.160 |
|  | (0.19) | (0.19) | (0.19) |
| Lived Alone at t_1_ | 0.010 | 0.010 | 0.007 |
|  | (0.07) | (0.07) | (0.07) |
| Received Formal Care at t_1_ | -0.071 | -0.071 | -0.062 |
|  | (0.10) | (0.10) | (0.10) |
| Received Formal Care at t_2_ | -0.063 | -0.060 | -0.065 |
|  | (0.08) | (0.08) | (0.08) |
| Rural Residence | 0.005 | 0.005 | 0.006 |
|  | (0.06) | (0.06) | (0.06) |
| Year Dummies (ref. = 2012) |  |  |  |
| 2014 | -0.072 | -0.072 | -0.074 |
|  | (0.06) | (0.06) | (0.06) |
| 2016 | -0.177** | -0.178** | -0.178** |
|  | (0.06) | (0.06) | (0.06) |
| 2018 | -0.152* | -0.153* | -0.152* |
|  | (0.06) | (0.06) | (0.06) |
| Constant | 12.547** | 12.566** | 12.726** |
|  | (4.30) | (4.30) | (4.30) |
| **N** | **8332** | **8332** | **8332** |

* p<.05, ** p<.01, *** p<.001

**Supplementary Table 5.** Mixed-effect regressions predicting depressive symptoms at t_2_, analyzing the heterogeneity by formal care receipts (n = 8,332).

|  | **Model 5.1** | **Model 5.2** |
| --- | --- | --- |
|  | Heterogeneity by  t_1_ Informal Care Receipt | Heterogeneity by  t_2_ Informal Care Receipt |
| Informal Care Instability (ref. = No Informal Care Receipt over Time) | |  |
| Persistent Care | -0.331*** | -0.320*** |
|  | (0.07) | (0.07) |
| Care Addition | -0.102 | -0.087 |
|  | (0.07) | (0.07) |
| Total Care Loss | -0.155 | -0.165 |
|  | (0.08) | (0.09) |
| Partial Care Loss | -0.322** | -0.302** |
|  | (0.10) | (0.11) |
| Care Transition | -0.136 | -0.034 |
|  | (0.13) | (0.13) |
| Receiving Formal Care at t_1_ (FC_1_) | 0.127 |  |
|  | (0.24) |  |
| FC_1_ * Persistent Care | -0.287 |  |
|  | (0.27) |  |
| FC_1_ * Care Addition | -0.372 |  |
|  | (0.29) |  |
| FC_1_ * Total Care Loss | 0.069 |  |
|  | (0.34) |  |
| FC_1_ * Partial Care Loss | -0.107 |  |
|  | (0.41) |  |
| FC_1_ * Care Transition | -0.435 |  |
|  | (0.42) |  |
| Receiving Formal Care at t_2_ (FC_2_) |  | 0.202 |
|  |  | (0.19) |
| FC_2_ * Persistent Care |  | -0.322 |
|  |  | (0.23) |
| FC_2_ * Care Addition |  | -0.480 |
|  |  | (0.25) |
| FC_2_ * Total Care Loss |  | 0.076 |
|  |  | (0.27) |
| FC_2_ * Partial Care Loss |  | -0.241 |
|  |  | (0.28) |
| FC_2_ * Care Transition |  | -0.913** |
|  |  | (0.34) |
| No. of ADL Limitations at t_2_ | 0.229*** | 0.231*** |
|  | (0.02) | (0.02) |
| No. of IADL Limitations at t_2_ | 0.193*** | 0.196*** |
|  | (0.02) | (0.02) |
| Age | -0.095*** | -0.095*** |
|  | (0.03) | (0.03) |
| Age Squared | 0.000* | 0.000* |
|  | (0.00) | (0.00) |
| Female | 0.177*** | 0.178*** |
|  | (0.05) | (0.05) |
| Race (ref. = White) |  |  |
| Black | -0.148* | -0.149* |
|  | (0.06) | (0.06) |
| Latino | 0.123 | 0.122 |
|  | (0.07) | (0.07) |
| Other | -0.019 | -0.022 |
|  | (0.13) | (0.13) |
| Educational Attainment (ref. = Less than High School) |  |  |
| High School Graduate | -0.058 | -0.059 |
|  | (0.06) | (0.06) |
| Some College | -0.166* | -0.164* |
|  | (0.07) | (0.07) |
| BA Degree | -0.338*** | -0.338*** |
|  | (0.09) | (0.09) |
| CES-D at t_1_ (ref. = 0-1) |  |  |
| 2-4 | 1.071*** | 1.071*** |
|  | (0.05) | (0.05) |
| 5-8 | 2.652*** | 2.647*** |
|  | (0.06) | (0.06) |
| Missing due to Proxy Interview | 0.915*** | 0.910*** |
|  | (0.19) | (0.19) |
| No. of Chronic Disease at t_1_ | 0.093*** | 0.092*** |
|  | (0.02) | (0.02) |
| No. of ADL Limitations at t_1_ | -0.045** | -0.047** |
|  | (0.02) | (0.02) |
| No. of IADL Limitations at t_1_ | -0.001 | -0.005 |
|  | (0.02) | (0.02) |
| Marital Status at t_1_ (ref. = Married/Partnered) |  |  |
| Divorced/Separated | 0.177* | 0.178* |
|  | (0.07) | (0.07) |
| Widowed | 0.027 | 0.027 |
|  | (0.08) | (0.08) |
| Never Married | 0.006 | 0.005 |
|  | (0.11) | (0.11) |
| Number of Proximate Children at t_1_ | -0.025 | -0.025 |
|  | (0.02) | (0.02) |
| Spouse Dead between t_1_ and t_2_ | 0.714*** | 0.709*** |
|  | (0.12) | (0.12) |
| Divorced/Separated between t_1_ and t_2_ | 0.156 | 0.156 |
|  | (0.19) | (0.19) |
| Lived Alone at t_1_ | 0.004 | 0.001 |
|  | (0.07) | (0.07) |
| Logged Household Wealth at t_1_ | -0.456 | -0.434 |
|  | (0.28) | (0.28) |
| Rural Residence | 0.004 | 0.004 |
|  | (0.06) | (0.06) |
| Year Dummies (ref. = 2012) |  |  |
| 2014 | -0.070 | -0.076 |
|  | (0.06) | (0.06) |
| 2016 | -0.177** | -0.181** |
|  | (0.06) | (0.06) |
| 2018 | -0.150* | -0.157** |
|  | (0.06) | (0.06) |
| Constant | 12.784** | 12.440** |
|  | (4.30) | (4.30) |
| **N** | **8332** | **8332** |
| * p<.05, ** p<.01, *** p<.001 |  |  |

**Supplementary Table 6.** Mixed-effect regressions predicting depressive symptoms at t_2_, adjusting for inverse probability weights for sample attrition (n = 8,332).

|  | **Model 6.1** | **Model 6.2** | **Model 6.3** |
| --- | --- | --- | --- |
|  | Direct Effects of Care Stability | Interactions with ADL Limitations | Interactions with IADL Limitations |
| Informal Care Instability (ref. = No Informal Care Receipt over Time) | |  |  |
| Persistent Care | -0.351*** | -0.317** | -0.267** |
|  | (0.07) | (0.10) | (0.08) |
| Care Addition | -0.121 | -0.011 | 0.047 |
|  | (0.07) | (0.11) | (0.10) |
| Total Care Loss | -0.139 | -0.236 | -0.122 |
|  | (0.08) | (0.12) | (0.10) |
| Partial Care Loss | -0.328** | -0.392** | -0.114 |
|  | (0.10) | (0.15) | (0.15) |
| Care Transition | -0.169 | 0.097 | -0.009 |
|  | (0.13) | (0.22) | (0.22) |
| No. of ADL Limitations at t_2_ (ADL) | 0.194*** | 0.217*** | 0.198*** |
|  | (0.02) | (0.04) | (0.02) |
| ADL * Persistent Care |  | -0.025 |  |
|  |  | (0.04) |  |
| ADL * Care Addition |  | -0.058 |  |
|  |  | (0.05) |  |
| ADL * Total Care Loss |  | 0.059 |  |
|  |  | (0.06) |  |
| ADL * Partial Care Loss |  | 0.015 |  |
|  |  | (0.06) |  |
| ADL * Care Transition |  | -0.124 |  |
|  |  | (0.09) |  |
| No. of IADL Limitations at t_2_ (IADL) | 0.234*** | 0.236*** | 0.394*** |
|  | (0.02) | (0.02) | (0.06) |
| IADL * Persistent Care |  |  | -0.165* |
|  |  |  | (0.07) |
| IADL * Care Addition |  |  | -0.215** |
|  |  |  | (0.07) |
| IADL * Total Care Loss |  |  | -0.064 |
|  |  |  | (0.09) |
| IADL * Partial Care Loss |  |  | -0.235** |
|  |  |  | (0.09) |
| IADL * Care Transition |  |  | -0.200 |
|  |  |  | (0.12) |
| Age | -0.095*** | -0.094*** | -0.094*** |
|  | (0.03) | (0.03) | (0.03) |
| Age Squared | 0.000* | 0.000* | 0.000* |
|  | (0.00) | (0.00) | (0.00) |
| Female | 0.177*** | 0.180*** | 0.180*** |
|  | (0.05) | (0.05) | (0.05) |
| Race (ref. = White) |  |  |  |
| Black | -0.150* | -0.154* | -0.153* |
|  | (0.06) | (0.06) | (0.06) |
| Latino | 0.123 | 0.120 | 0.117 |
|  | (0.08) | (0.08) | (0.08) |
| Other | -0.020 | -0.031 | -0.027 |
|  | (0.12) | (0.12) | (0.12) |
| Educational Attainment (ref. = Less than High School) |  |  |  |
| High School Graduate | -0.052 | -0.053 | -0.057 |
|  | (0.06) | (0.06) | (0.06) |
| Some College | -0.157* | -0.157* | -0.159* |
|  | (0.07) | (0.07) | (0.07) |
| BA Degree | -0.327*** | -0.327*** | -0.332*** |
|  | (0.09) | (0.09) | (0.09) |
| Logged Household Wealth at t_1_ | -0.455 | -0.469 | -0.463 |
|  | (0.27) | (0.27) | (0.27) |
| CES-D at t_1_ (ref. = 0-1) |  |  |  |
| 2-4 | 1.092*** | 1.089*** | 1.089*** |
|  | (0.06) | (0.06) | (0.06) |
| 5-8 | 2.697*** | 2.692*** | 2.699*** |
|  | (0.07) | (0.07) | (0.07) |
| Missing due to Proxy Interview | 0.930*** | 0.928*** | 0.936*** |
|  | (0.21) | (0.21) | (0.21) |
| No. of Chronic Disease at t_1_ | 0.094*** | 0.094*** | 0.093*** |
|  | (0.02) | (0.02) | (0.02) |
| No. of ADL Limitations at t_1_ | -0.045* | -0.047** | -0.045* |
|  | (0.02) | (0.02) | (0.02) |
| No. of IADL Limitations at t_1_ | -0.002 | -0.001 | -0.002 |
|  | (0.02) | (0.02) | (0.02) |
| Marital Status at t_1_ (ref. = Married/Partnered) |  |  |  |
| Divorced/Separated | 0.177* | 0.172* | 0.169* |
|  | (0.08) | (0.08) | (0.08) |
| Widowed | 0.030 | 0.028 | 0.027 |
|  | (0.08) | (0.08) | (0.08) |
| Never Married | 0.018 | 0.016 | 0.019 |
|  | (0.10) | (0.10) | (0.10) |
| Number of Proximate Children at t_1_ | -0.026 | -0.025 | -0.024 |
|  | (0.02) | (0.02) | (0.02) |
| Spouse Dead between t_1_ and t_2_ | 0.720*** | 0.711*** | 0.711*** |
|  | (0.13) | (0.13) | (0.13) |
| Divorced/Separated between t_1_ and t_2_ | 0.160 | 0.143 | 0.140 |
|  | (0.22) | (0.22) | (0.22) |
| Lived Alone at t_1_ | 0.006 | 0.003 | -0.005 |
|  | (0.07) | (0.07) | (0.07) |
| Received Formal Care at t_1_ | -0.074 | -0.070 | -0.086 |
|  | (0.10) | (0.10) | (0.10) |
| Received Formal Care at t_2_ | -0.060 | -0.071 | -0.073 |
|  | (0.08) | (0.08) | (0.08) |
| Rural Residence | 0.002 | -0.001 | -0.003 |
|  | (0.06) | (0.06) | (0.06) |
| Year Dummies (ref. = 2012) |  |  |  |
| 2014 | -0.076 | -0.075 | -0.080 |
|  | (0.06) | (0.06) | (0.06) |
| 2016 | -0.179** | -0.178** | -0.183** |
|  | (0.06) | (0.06) | (0.06) |
| 2018 | -0.154* | -0.154* | -0.159** |
|  | (0.06) | (0.06) | (0.06) |
| Constant | 12.738** | 12.897** | 12.733** |
|  | (4.18) | (4.19) | (4.20) |

* p<.05, ** p<.01, *** p<.001
